# Editorial: Volume II: Tumor microenvironment in cancer hallmarks and therapeutics

**DOI:** 10.3389/fmolb.2023.1182083

**Published:** 2023-03-28

**Authors:** Rongchen Shi, Yuan Gao, José Alexandre Ferreira, Na Luo, Hongming Miao

**Affiliations:** ^1^ Frontier Medical Training Brigade, Third Military Medical University (Army Medical University), Xinjiang, China; ^2^ Department of Pathophysiology, College of High Altitude Military Medicine, Third Military Medical University (Army Medical University), Chongqing, China; ^3^ Experimental Pathology and Therapeutics Group, Research Center of IPO Porto (CI-IPOP)/RISE@CI-IPOP (Health Research Network), Portuguese Oncology Institute of Porto (IPO-Porto)/Porto Comprehensive Cancer Center Raquel Seruca (Porto.CCC), Porto, Portugal; ^4^ Department of Anatomy and Histology, School of Medicine, Nankai University, Tianjin, China

**Keywords:** cancer biomarkers, tumor microenvironment, cancer immunotherapy, diagnosis, prognosis

Cancer biomarkers have many important applications in oncology, including risk assessment, diagnosis, prognosis, prediction of treatment response, and disease progression (Sarhadi and Armengol, 2022). In the past, cancer biomarkers were mainly focused on the discovery of mutated genes occurring in cancer cells themselves (Jahangiri and Aghi, 2012; Ganguly et al., 2019). However, tumor cells live in the immunosuppressed tumor microenvironment, and the immune response is also critical to the prognosis of patients. Therefore, there is an urgent need to explore new cancer biomarkers, which could not only indicate prognosis, but also guide tumor therapy, especially tumor immunotherapy (Wu and Dai, 2017; Liu et al., 2022).

This Research Topic is dedicated to publishing new prognostic and therapeutic biomarkers, especially those related to tumor microenvironment (TME), based on large databases and large sample analyses, which will be more beneficial for tumor screening and treatment. A total of 13 articles are included in this Research Topic.

Five articles are devoted to single gene database analysis. Du et al. used the database for somatic mutation analysis and found that FRAS1 Related Extracellular Matrix 2 (FREM2) was one of the genes with the highest mutation frequency in patients with colorectal adenocarcinoma and was closely associated with poor prognosis. Meanwhile, the random forest method was used to construct a prognosis model with good predictive function based on FREM2 mutation. The prediction accuracy was high (83.9%), and a total of 13 prognostic pattern characteristic genes related to overall survival were identified. These results suggest that FREM2 mutation may be a potential prognostic marker for colon cancer. Another research found that collagen triple helix repeat containing 1 (CTHRC1), a glycosylated protein, was significantly expressed in cancer tissues of colorectal adenocarcinoma patients and was associated with poor patient outcomes. More importantly, authors found that CTHRC1 expression was positively correlated with a variety of immune cell infiltration, including CD8^+^ T cells, CD4^+^ T cells, neutrophils, macrophages, and dendritic cells, which may provide a theoretical basis for the development of new immunotherapeutic targets based on CTHRC1. Liu et al. found that interleukin-1 receptor-associated kinases1 (IRAK1), an active kinase that plays a key role in the IL-1/TLR signaling pathway, is upregulated in more than two dozen cancers. And the expression level of IRAK1 is closely related to the efficacy of anti-PDL1, which may be a reliable marker for predicting the efficacy of tumor immunotherapy. In addition, pan-cancer analysis of targeted type 2 mannose receptor C (MRC2) suggested significant associations with immune cell infiltration, immunomodulators, and immunotherapeutic markers, particularly in patients with metastatic melanoma and advanced urothelial carcinoma. Bai et al. also found that the expression of laminin subunit gamma 1 (LAMC1) in different types of renal cancer was completely opposite to the prognosis, and database analysis suggested that this might be closely related to the different tumor immune microenvironment.

Another five papers are devoted to multigene database analysis. A retrospective study of breast cancer samples by Zhu et al. showed that the Systemic Inflammation Index (SIRI) can independently predict breast cancer survival. Lower SIRI predicted increased disease-free survival and overall survival. In addition, Sun et al. developed a considerable nomogram. Trp-related immune gene (TRIG) scores were negatively correlated with immune activation and overall survival. At the same time, TRIG score was also significantly correlated with immune cell infiltration and immune checkpoint expression in TME, which might provide new strategies for prognosis assessment and tumor immunotherapy in lung adenocarcinoma patients. Ephrin family genes (EFNs) and the prognostic and immunological characteristics of liver cancer patients were analyzed in another research. The authors found that EFNA3, EFNA4 and EFNB1 were independent prognostic factors and were closely related to tumor immunity. Zhou et al. conducted a comprehensive analysis of the relationship between genes related to single carbon metabolism and prognosis, chemotherapy resistance and immunotherapy in patients with lung adenocarcinoma. According to the expression of 7 prognostic related genes in 497 LUAD samples, the authors divided the sample into two clusters, and pointed out that cluster 1 had worse prognosis and stronger chemotherapy resistance, but cluster 1 had more significant immunotherapy efficacy, providing a theoretical basis for immunotherapy in LUAD patients. In a study of the association between cuproptosis-related genes and papillary renal cell carcinoma (PRCC) development, prognosis, and treatment, authors created and validated a risk score for predicting overall survival, indicating that the lower the risk score, the better the tumor immune microenvironment, the longer the overall survival, and the stronger the sensitivity to chemotherapy drugs.

In addition, Liu et al. analyzed the tumor immune microenvironment and found that the survival rate of cervical cancer patients with low immune level in tumor was lower than that of patients with high immune level, which may be related to the reduced level of immune infiltration caused by the high methylation level in the TME. This finding is of great significance for hierarchical management of patients and precise targeted therapy.

Finally, two reviews are included in this Research Topic. One paper summarized the role of cytokines most associated with EMT in tumor progression, invasion, migration, and metastasis formation of bladder cancer. The other provides a comprehensive discussion of exosomes and their role in various aspects of cancer biology. Both these cytokines and exosomes might serve as new biomarkers for efficient diagnosis.

In conclusion, recent studies have used large databases and other state-of-art technologies to accurately analyze the significance of one or more genes that are misregulated in a specific TME for tumor diagnosis, prognosis, and treatment. These studies have greatly expanded our current understanding of tumor biomarkers and will facilitate further development.
